# Enhanced Modification of Fast-Growing Wood: Application and Evaluation of Castor Oil-Based Unsaturated Polyester Resin

**DOI:** 10.1021/acsomega.3c07565

**Published:** 2023-11-13

**Authors:** Tianle Xu, Xinran Ju, Hui Tang, Wenli Xiang, Zhiliang Wang, Yandi Li

**Affiliations:** † Faculty of Chemical Engineering, Kunming University of Science and Technology, Kunming 650093, China; ‡ Faculty of Science, University of Sydney, Camperdown, New South Wales 2050, Australia

## Abstract

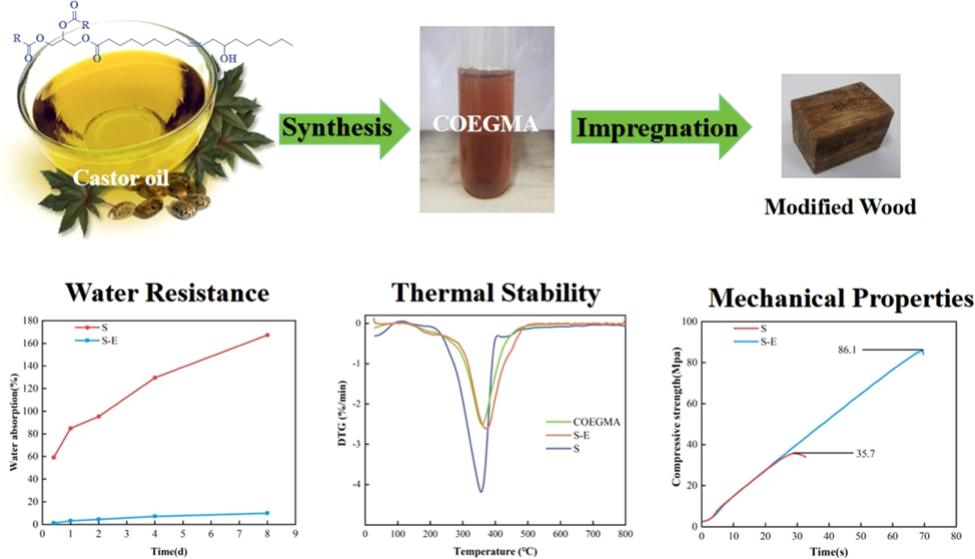

A type of multifunctional maleic acid ester monomer (COEGMA) was synthesized using castor oil as raw material, and green wood–plastic composites were prepared by chemically impregnating and curing the monomer into wood. The structure of the synthesized products at various stages was determined by FT-IR spectroscopy, ^1^H NMR, and GPC, and the curing experimental conditions were optimized. The results show that the water absorption of wood–plastic composites treated with COEGMA is reduced from the original 167.3% to less than 20%. The compressive strength has increased from 35.7 to 86.1 MPa, and the thermal stability has also increased by 40 °C. This research provides promising prospects for the development of environmentally friendly wood–plastic composites, especially as fossil resources become scarce and environmental pollution becomes more severe.

## 1 Introduction

Wood is widely used in contemporary home construction and furniture production due to its high mechanical strength, insulation, and soundproofing properties.^1−5^ However, native wood has large voids that can absorb significant amounts of small molecules such as water vapor during use, causing deformation of the wood’s appearance and reducing its strength and durability, thereby limiting its application.^6,7^ Heat treatment is generally considered an effective and simple method for modifying wood properties. However, high temperatures can increase the brittleness of wood products, chemical structural changes, and degradation of polysaccharides.^8−11^ Another effective method for enhancing wood properties is chemical modification, which involves the functional improvement of fast-growing wood to obtain performance-enhanced wood–plastic composites,^12^ which has attracted widespread attention and research.

Unsaturated polyester resin (UPR) based on petroleum has traditionally been widely used as a thermosetting polymer. However, due to its predominantly nonrenewable fossil resources, which are gradually depleting, serious environmental pollution, such as microplastics and marine pollution, and expensive prices, using biobased materials to replace nonrenewable resources and prepare biobased wood–plastic composites is necessary for sustainable development.^13−15^

The widespread availability and relatively high cost-effectiveness of vegetable oils make them one of the most important sustainable resources in the chemical industry.^16−18^ The main component is triglycerides, which have a long chain structure and characteristic functional groups that can react with anhydrides, making them rich, renewable, and widely used polymer materials.^19^ Das et al.^20^ used tung oil to prepare a new type of unsaturated polyester resin, which has higher impact strength, creep resistance, modulus, and hardness. Costa et al.^21^ studied a new type of unsaturated polyester resin based on soybean oil and coconut oil, which has considerable thermal stability. Other researchers have used different raw materials to enhance the mechanical properties of unsaturated polyester resins, such as hexamethylene diisocyanate (HDI) trimer,^22^ bismaleimide,^23^ and isophorone diisocyanate (IPDI).^24^ Some scholars have also injected synthesized epoxy oligomers into wood to prepare wood–plastic composites.^25^ Impregnating wood with low-molecular-weight monomers or precursors has been proven to be an effective method for improving wood dimensional stability and durability.^26,27^ Unsaturated monomers or thermosetting resins are usually used to stabilize or enhance wood because they can polymerize or cross-link well in wood.

Castor oil (CO) is extracted from the seeds of *Ricinus communis* plants and is widely used in the manufacture of soaps, cosmetics, paints, dyes, plastics, medicines, perfumes, and other products.^30,31^ It contains a high content of ricinoleic acid, and the hydroxyl groups in it are active sites for derivatization. These active sites can be used to obtain multifunctional castor oil-based acrylic acid esters through chemical modification, which have more active C=C groups compared to other plant oil-based acrylic acid esters. For instance, Rao et al.^32^ synthesized a trifunctional acrylic ester diluent by reacting castor oil with diethanolamine and then with acryloyl chloride (AC). However, the use of amine reagents with unpleasant odors can cause serious irritation or corrosion to human biological tissues such as skin and the respiratory tract.

Impregnating wood with styrene can delay wood discoloration, but the degradation of styrene during wood decay (aging of styrene in outdoor applications, particularly under light and heat) limits its use in high-risk areas.^28^ Unsaturated polyester resin is a commonly used thermosetting material that can effectively improve the bending strength and elastic modulus of wood, thereby prolonging its service life.^29^ However, to the best of our knowledge, few studies have applied biobased unsaturated polyester resins to wood reinforcement modification in published articles. The aim of this experiment is to combine biobased unsaturated polyester with wood to prepare a biobased wood–plastic composite material, enhancing the service life of fast-growing wood and expanding its range of applications.

**Figure 1:**
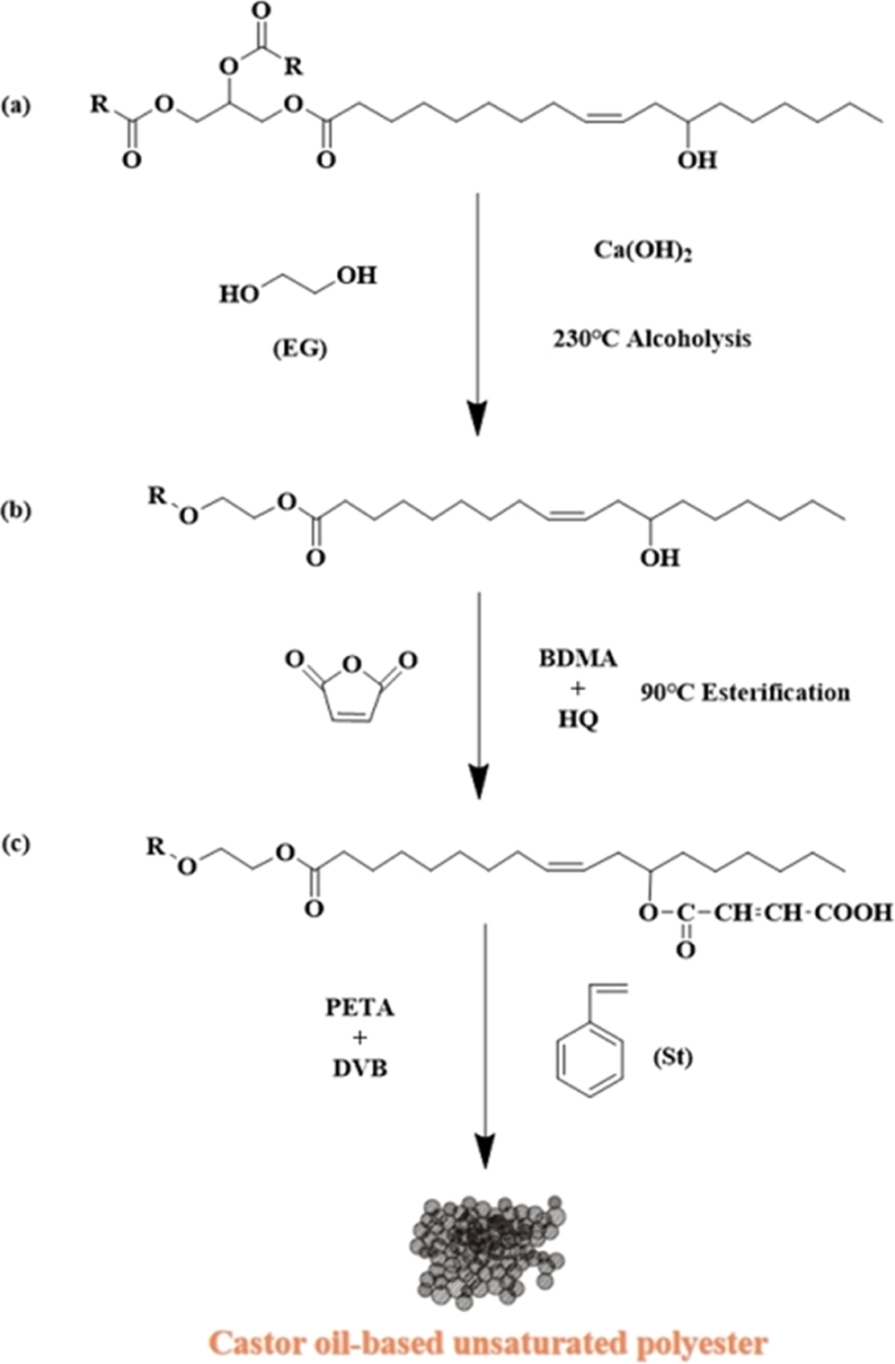
Synthesis of CO-UPR.

In this study, a multifunctional maleic acid ester monomer (COEGMA) was synthesized by reacting castor oil with glycol and maleic anhydride, and a three-dimensional network structure of castor oil-based unsaturated polyester resin (CO-UPR) was obtained by adding a small amount of diluent and cross-linking agent (Figure 1). The chemical impregnation method was used to modify the Chinese fir (Figure 7), and its properties, such as water resistance, dimensional stability, and thermal stability, were studied.

## 2 Experimental Section

### 2.1 Main Raw Materials and Instruments

#### 2.1.1. Experimental Materials and Reagents

Both castor oil and fir are purchased in the market; the alcoholysis agent ethylene glycol (EG, A.R.) was purchased from Guangdong Guanghua Technology Co., Ltd., China; catalyst calcium hydroxide (A.R.) and N,N-dimethylbenzylamine (BDMA, A.R.) was purchased from Xilong Science Co., Ltd., China; maleic anhydride (MA, A.R.) was purchased from Shanghai Macklin Biochemical Technology Co., Ltd., China; curing initiator benzoyl peroxide (BPO, A.R.) and polymerization inhibitor hydroquinone (HQ, A.R.) were provided by Sinopharm Chemical Reagent Co., Ltd., China; the diluent monomer styrene (St, A.R.) was produced by Shanghai McLean Biochemical Science Co., Ltd., China; and the cross-linking agents divinylbenzene (DVB, A.R.) and pentaerythritol triacrylate (PETA, A.R.) were purchased from Tianjin Baima Technology Co., Ltd., China.

#### 2.1.2. Analytical Testing Instruments

Analytical testing instruments include the following: Rotational viscometer: NDJ-1 type, Shanghai Precision Scientific Instrument Co., Ltd.; Fourier transform infrared spectrometer (FT-IR): Spectrum Two, PerkinElmer; Pressure testing machine, YAW-100D, Jinan Zhongluchang Testing Machine Manufacturing Co., Ltd.; thermogravimetric analyzer (TGA): TGA-50, Shimadzu Corporation, Japan; X-ray photoelectron spectrometer (XPS): PHI5000 Versaprobe-II, Ulvac-Phi, Japan; Scanning electron microscope (SEM): Model TESCAN VEGA 3, Czech Tescan Company; Gel chromatograph, LC98IIRI, Beijing Temperature Analysis Instrument Technology Development Co., Ltd.

### 2.2 Preparation of Modified Wood Specimens

#### 2.2.1. Synthesis of Castor Oil-Based Unsaturated Polyester

**Figure 2:**
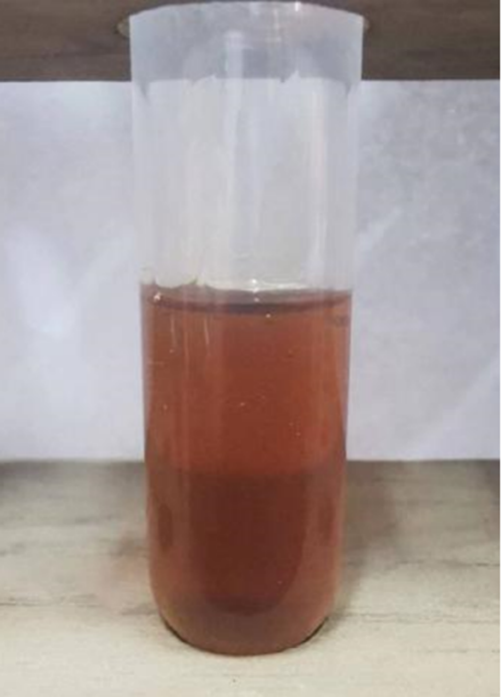
Synthesized multifunctional maleic acid ester monomers COEGMA.

Castor oil and ethylene glycol were added at a molar ratio of 1:2, and 0.5 wt % of calcium hydroxide was added to a three-necked flask. It was stirred and heated to 230 °C. After a constant temperature reaction for 2 h, it was cooled to 70 °C and maleic anhydride (the number of moles added is the same as that of the hydroxyl groups in the system) was added. It was stirred for 30 min, and 1 wt % of N,N-dimethylbenzylamine was added after the reactants were fully mixed. The temperature was increased to 90 °C,^33^ and the acid value was monitored according to GB/T 6743-2008.^34^ When the acid value drops to 230 mgKOH/g approximately, the diluent styrene (with a mass ratio of 1:3 styrene to oligomer) and cross-linker were added.^35,36^ When the UPR oligomer was completely dissolved in the diluent/cross-linker, the stirring was stopped to prepare the biobased UPR (Figure 2).

#### 2.2.2. Modification of Wood Specimens

To ensure uniformity among the wood samples (20 × 20 × 30 mm^3^), Chinese fir specimens were chosen from a consistent board, ensuring each piece was intact with no imperfections and had a density approximating the average of 0.36 ± 0.03 g/cm^3^. These specimens were dried in a vacuum at 103 ± 2 °C for a duration of 2 h before being placed into the impregnation container. The container was then pre-evacuated to a pressure of −0.08 MPa for 0.5 h, followed by the introduction of the UPR impregnation solution. This vacuum level was maintained for another 30 min. Subsequently, the samples were allowed to remain submerged in the solution at atmospheric pressure for an additional 2 h to ensure thorough immersion.^33^ Post this process, the samples were oven-baked at 100 °C for 2 h, finalizing the wood’s modification.

### 2.3 Test Analysis Method

#### 2.3.1. Determination of Curing Degree of Dipping System

Assessing the curing level of a pure UPR impregnation system: based on the approach outlined in GB/T 2576-2005,^37^ the soluble elements in the postcured UPR system are extracted using ethyl acetate near its boiling point with a Soxhlet extractor. The components that do not dissolve are viewed as cured resin. Formula 1 is used to compute the curing degree, denoted as α
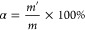
1*m* represents the sample’s weight before the extraction process, measured in mg. *m*′ stands for the sample’s weight post extraction, also in mg.

Evaluating the curing level of the UPR system within wood: using the method for determining the curing degree of a pure UPR impregnation system as a guide, the curing degree, labeled as β, for the UPR system integrated into wood is determined using Formula 2
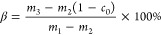
2where *m*_1_ denotes the weight of the altered wood sample before extraction, measured in mg. *m*_2_ signifies the weight of the log present in the altered wood sample before extraction, also in mg. *m*_3_ represents the weight of the altered wood sample after the extraction process in mg. *c*_0_ refers to the percentage loss rate of the log’s mass post extraction.

For this procedure, 5 samples are chosen.

Assessing the impregnation rate and the increase in weight due to curing in modified wood.

The UPR system’s impregnation rate into the wood, labeled as IY, is determined by using Formula 3. Additionally, the rate of weight gain from the curing process, denoted as WPG, is computed by using Formula 4
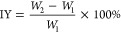
3
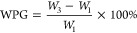
4*W*_1_ is the mass of the log specimen, g; *W*_2_ is the mass of the wood specimen after impregnation, g; and *W*_3_ is the mass of the wood specimen after curing, g. The number of selected samples is 5.

#### 2.3.2. Determination of Water Absorption Resistance of Modified Wood

According to the measurement method in GB/T 1934.1–2009,^38^ the measurement period is 7 days, and the water absorption rate A of the modified wood specimen is calculated according to Formula 5
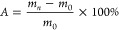
5

In the given equation, *m*_0_ refers to the weight of the wood sample when it is fully dried, measured in grams. Meanwhile, *m_n_* represents the weight of the wood sample after it has absorbed water, also in grams.

For the modified wood sample, its resistance to water absorption, termed as WRE, is determined using Formula 6
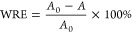
6

Within the equation, *A*_0_ symbolizes the percentage of water absorption in the log sample, and *A* denotes the water absorption rate for the modified wood sample.

For this procedure, 5 samples have been chosen.

#### 2.3.3. Determination of Compressive Strength along the Grain of Modified Wood

Following the procedure outlined in GB/T 1935–2009,^39^ a pressure-testing device was employed to gauge the compressive strength of the wood samples along the grain. The sample was ensured that it was centered on the spherical movable support of the test machine and aligned with the grain’s direction. The load speed was set at 0.5 KN/s, and the load at which the specimen fails was noted. The compressive strength (σ, in MPa) of the modified wood sample was determined using Formula 7
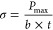
7

In the given equation, *P*_max_ denotes the load at which the specimen fails, measured in Newtons (N). The width of the specimen is represented by *b* in millimeters (mm), and *t* stands for the specimen’s length, also in millimeters (mm).

#### 2.3.4. FT-IR Spectroscopy

FT-IR spectroscopy was used to characterize the castor oil hydrolysis products, esterification products, and modified wood samples. We used attenuated total reflection (ATR) spectroscopy with 8 reflection scans for detection, and the test wavenumber range was 4000–400 cm^–1^.

#### 2.3.5. Proton Nuclear Magnetic Resonance (^1^H NMR) Analysis

Deuterated acetone was used as the solvent, and when the magnetic field was set to 400 M, the alcoholysis products and esterification products of castor oil were analyzed using a Bruker Avance 400 nuclear magnetic resonance spectrometer.

#### 2.3.6. Scanning Electron Microscopy (SEM)

Both the unaltered log and the modified wood samples were sliced along the chord direction, producing chord section pieces measuring 5 × 5 × 2 mm^3^. Scanning electron microscopy (SEM) was then used to examine the surface microstructure of these specimens. To prevent charge buildup during examination, the samples were coated with a layer of gold. They were then analyzed under magnifications of 1000× and 5000×, using an accelerating voltage set to 20 KV.

#### 2.3.7. Gel Permeation Chromatography (GPC)

An LC98IIRI gel chromatograph was used with tetrahydrofuran as the mobile phase. Polystyrene was used as the standard sample, and the flow rate was set at 0.35 mL/min. Temperature control: the weight-average molecular weight, number-average molecular weight, and polydispersity coefficient of the samples were measured at 40 °C. The product concentration was approximately 5.0 mg/mL.

#### 2.3.8. X-ray Photoelectron Spectroscopy (XPS)

The specimens from the fir log and the modified wood were sliced along the chord direction to create chord sections measuring 5 × 5 × 2 mm^3^. These samples were then subjected to X-ray photoelectron spectroscopy (XPS) to examine their surface chemical elements. An Al anode served as the X-ray source. The power was set at 50 KW, and the pass energy was 46.95 eV. When capturing the XPS full spectrum and the high-resolution spectrum, step sizes of 0.8 and 0.2 eV were used, respectively. The binding energy was referenced to C 1s of alkyl carbon, specifically at 284.8 eV, which was employed for charge correction.

#### 2.3.9. Thermogravimetric Analysis (TGA)

The specimens were ground down by using a pulverizer until they reached a powdery consistency with particle sizes smaller than 0.2 mm. Subsequently, the thermal stability of the modified wood was investigated using thermogravimetric analysis (TGA). The flow of nitrogen was maintained at a rate of 20 mL/min. The sample was then subjected to a temperature increase, starting from room temperature and escalating to 800 °C, with a consistent heating rate of 10 °C/min.

## 3 Results and Discussion

### 3.1 FT-IR Analysis of Castor Oil and CO-UPR Products at Each Stage

**Figure 3:**
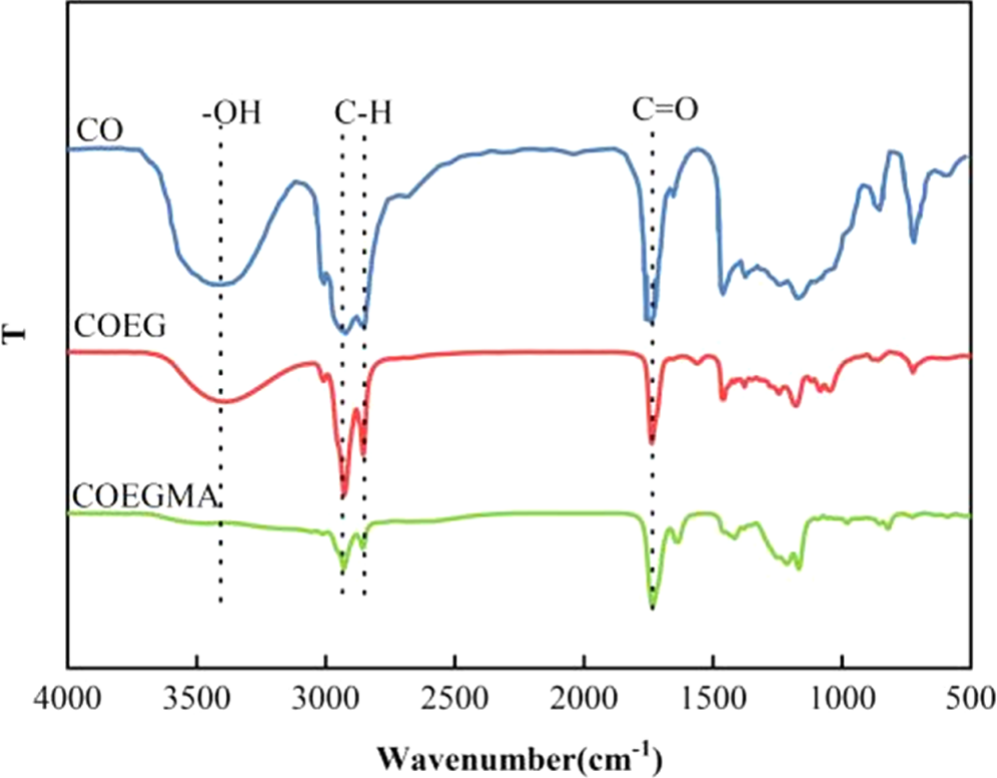
Infrared spectra of raw materials of unsaturated polyester resin and products of each stage of synthesis. In the figure, CO represents castor oil, COEG represents the alcoholysis product of castor oil, and COEGMA represents the esterification product.

The C–H bond symmetric and antisymmetric stretching vibration double absorption peaks around 2950 cm^–1^ of castor oil and CO-UPR products in each stage indicate the existence of alkyl chains. There is the strongest absorption peak at 1720 cm^–1^, which is the stretching vibration of the C=O bond, indicating the presence of an ester group; the broad and strong −OH bond absorption peak at around 3500 cm^–1^ and the absorption peak at 1230 cm^–1^ together indicate that this is the C–O bond stretching vibration of polyols^40^ (Figure 3). It was observed that the C–H stretching vibration absorption peak of the alkyl group in the castor oil structure and the C=O stretching vibration absorption peak of the ester group appeared at the same wavelength position of the castor oil alcoholysis product and the UPR oligomer spectra. It shows that some characteristic groups in the original castor oil structure are successfully introduced into the subsequent stage products during the alcoholysis of castor oil and the UPR synthesis. The esterification reaction proceeded as expected.

### 3.2 CO-UPR ^1^H NMR Analysis at Various Stages

**Figure 4:**
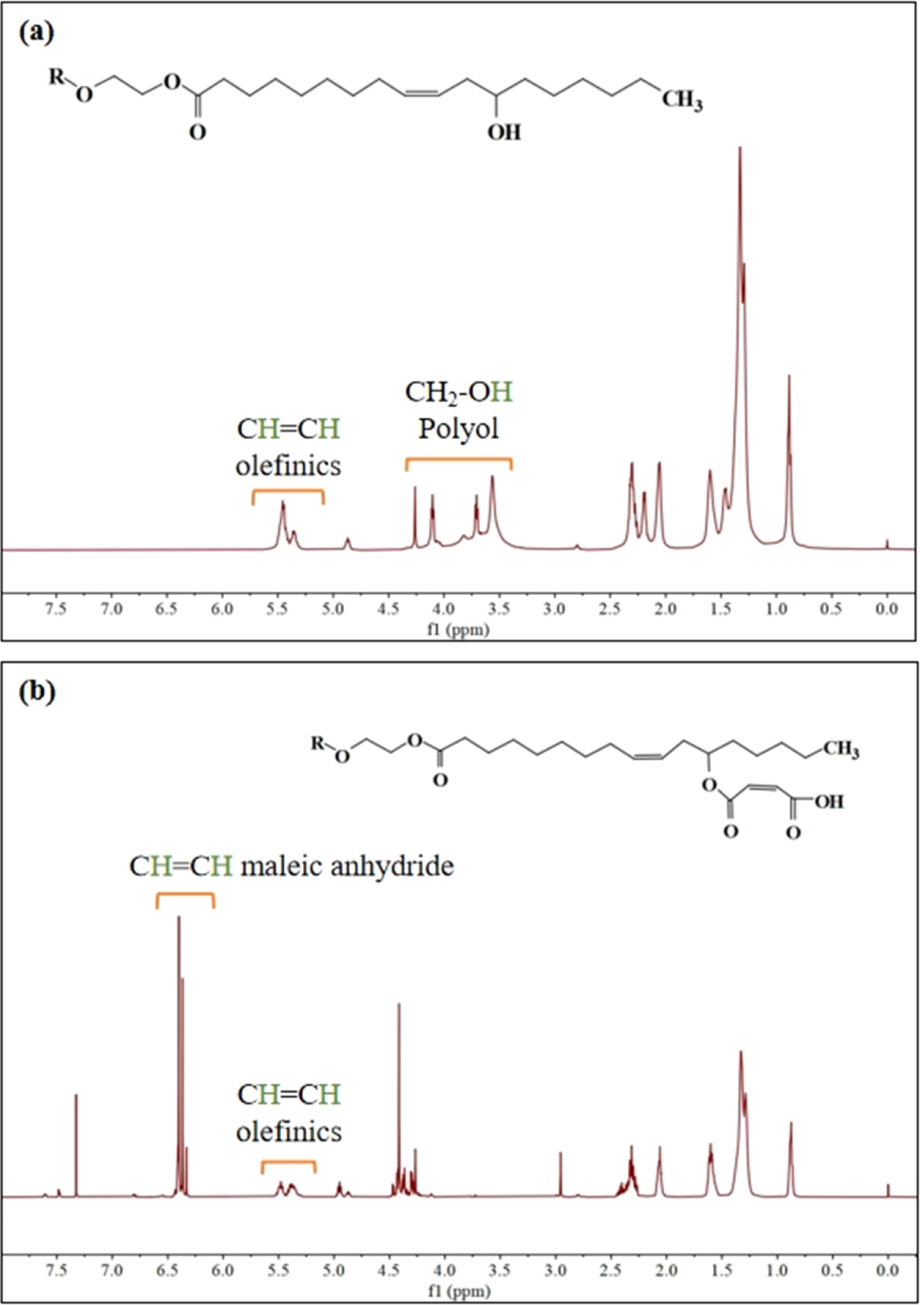
^1^H NMR analysis of products in the unsaturated polyester synthesis. Panel (a) represents the alcoholysis product of castor oil, and panel (b) represents the esterification product.

As Figure 4 shows, the signal at 0.88 ppm corresponds to the terminal methyl protons, the peak at 1.30–1.32 ppm is attributed to all of the internal CH_2_ groups in the fatty acid chain, and the unsaturation in the fatty acid chain is displayed at 5.36–5.46 ppm. Multiplets observed from 3.56 to 4.26 ppm correspond to protons connected to the OH group or CH_2_.^41^ According to Kumar et al.,^42^ monoglycerides were identified from other triglycerides through the multiplet analysis at 3.56–3.75 ppm. The disappearance of protons connected to the OH group or CH_2_, as well as the signal representing the maleic anhydride backbone (−CH=CH−) at 6.33–6.40 ppm,^43,44^ indicates the continuous consumption of the alkoxy group in the reaction. The expected esterification reaction proceeded smoothly.

### 3.3 GPC Analysis of Castor Oil-Based Unsaturated Polyester

**Figure 5:**
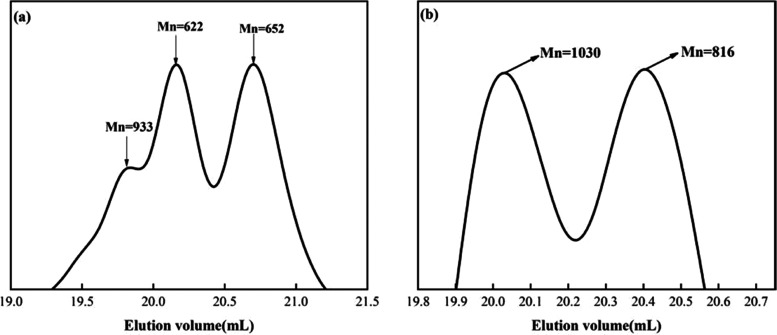
(a) GPC spectrogram of the product of castor oil after alcoholysis with ethylene glycol. (b) GPC spectrogram of the product after maleic anhydride esterification.

GPC analysis of the synthesized CO-UPR and its hydrolysis products can provide insight into the molecular weight and predicted structure of the products at the synthesis stage, further exploring their effects on the curing experiments. As shown in the figure, after castor oil was hydrolyzed with ethylene glycol, three peaks were observed with Mn values of 933, 653, and 622 (Figure 5a), respectively, corresponding to castor oil, the ester-exchanged hydrolysis product ethylene glycol ricinolate, and castor oil residue. After the maleic anhydride esterification of the hydrolysis product, two peaks were observed with Mn values of 1030 and 816 (Figure 5b), respectively, corresponding to COEGMA and castor oil maleate ester. It was found that ethylene glycol had strong hydrolysis ability in this case, but due to its only two hydroxyl groups, it cannot completely remove triglycerides, resulting in other components in the esterification products.

### 3.4 Curing Experiment of Castor Oil-Based Unsaturated Polyester

In order to explore the optimal conditions and feed ratios of CO-UPR curing, this paper adopts an orthogonal experiment (Table 1). Taking the curing degree of the synthetic product as a reference basis, it is used to investigate the influence of various factors on the synthetic product. The experimental results are shown in Table 2.

**Table 1:** Design Table for Curing Orthogonal Experiments

	factors			
level	type of cross-linking agent (A)	initiator dosage/% (B)	cross-linking agent dosage/% (C)	curing time/h (D)
1	DVB	1	1	2
2	DVB/PETA (1:1)	2	2	3
3	PETA	3	3	4

Note: the diluent is St, and the mass ratio of diluting with styrene is 3:1; the curing temperature is 100 °C.

**Table 2:** Orthogonal Table for Curing Orthogonal Experiment

	factors				
experiment number	A	B	C	D	curing degree (%)
1	1	1	1	1	85.1
2	1	2	2	2	83.2
3	1	3	3	3	86.3
4	2	1	2	3	81.7
5	2	2	3	1	87.9
6	2	3	1	2	85.3
7	3	1	3	2	81.2
8	3	2	1	3	83.2
9	3	3	2	1	85.9
Based on Curing Degree					
K1	254.6	248.0	253.5		258.8
K2	254.8	254.3	250.8		249.7
K3	250.4	257.5	255.4		251.2
k1	84.8	82.6	84.5		86.2
k2	84.9	84.7	83.6		83.2
k3	83.4	85.8	85.1		83.7
R	1.4	3.1	1.5		3.0

Influencing factors: Initiator dosage > curing time > cross-linking agent dosage > cross-linking agent type.

Optimal conditions: A2, B3, C3, D1.

The curing experiment under the optimal conditions selected by the orthogonal experiment resulted in a curing degree of 89.5%. It was found through experiments that although the synthesized UPR oligomer contained other components, it had little effect on the curing of CO-UPR. This is because the curing process of UPR is complex, and the C=C bonds on the linear unsaturated polyester molecular chain will undergo free-radical copolymerization with styrene, forming four types of cross-linking structures approximately: (I) cross-linking between molecules with the help of styrene; (II) cross-linking within the molecule with the help of styrene; (III) branching of the polyester molecule with the help of styrene; and (IV) styrene homopolymerization.^45^ In addition, steric hindrance effects lead to the presence of some unreacted unsaturated double bonds in the cured cross-linked network of the unsaturated polyester, making the network more disordered. Therefore, the other components in the UPR oligomer can also help CO-UPR to be further cured.

### 3.5 Reinforcing Properties of Wood Modified by UPR Impregnation

#### 3.5.1 Basic Property Analysis of Modified Wood

The influence of the UPR impregnation system on the reinforcement properties of modified wood is mainly reflected in the impregnation rate, curing weight gain rate, and water absorption resistance (Table 3).

**Table 3:** Properties of Modified Chinese Fir with CO-UPR Impregnation Systems

					*P*_s_				
UPR systems	viscosity (Pa·s)	wood type	Plog (g/cm^3^)	Pcuring (g/cm^3^)	outer	middle	inner	IY (%)	WPG (%)
CO-UPR	0.23 ± 0.02	S	0.41 ± 0.03	1.03 ± 0.23	1.14 ± 0.03	1.11 ± 0.011	1.09 ± 0.006	178.0 ± 0.08	170.3 ± 0.06

*P*_s_ is the density of the profile.

After impregnation of the spruce with the CO-UPR resin, both the impregnation rate and the curing weight gain rate reached over 170%. The differences in density between the outer, middle, and inner sections of the modified wood were not significant and were higher than the density of the fir log, decreasing gradually from the outer to the inner section. This indicates that the resin has successfully impregnated the interior of the wood, with a gradual decrease in the impregnation effect from the outer section to the inner section. However, the density differences were not significant due to the low viscosity of the CO-UPR impregnation system (0.23 Pa·s), which allowed the low viscosity impregnation fluid to better fill the gaps deep inside the wood and improve the modification effect, making it more suitable for industrial application.

**Figure 6:**
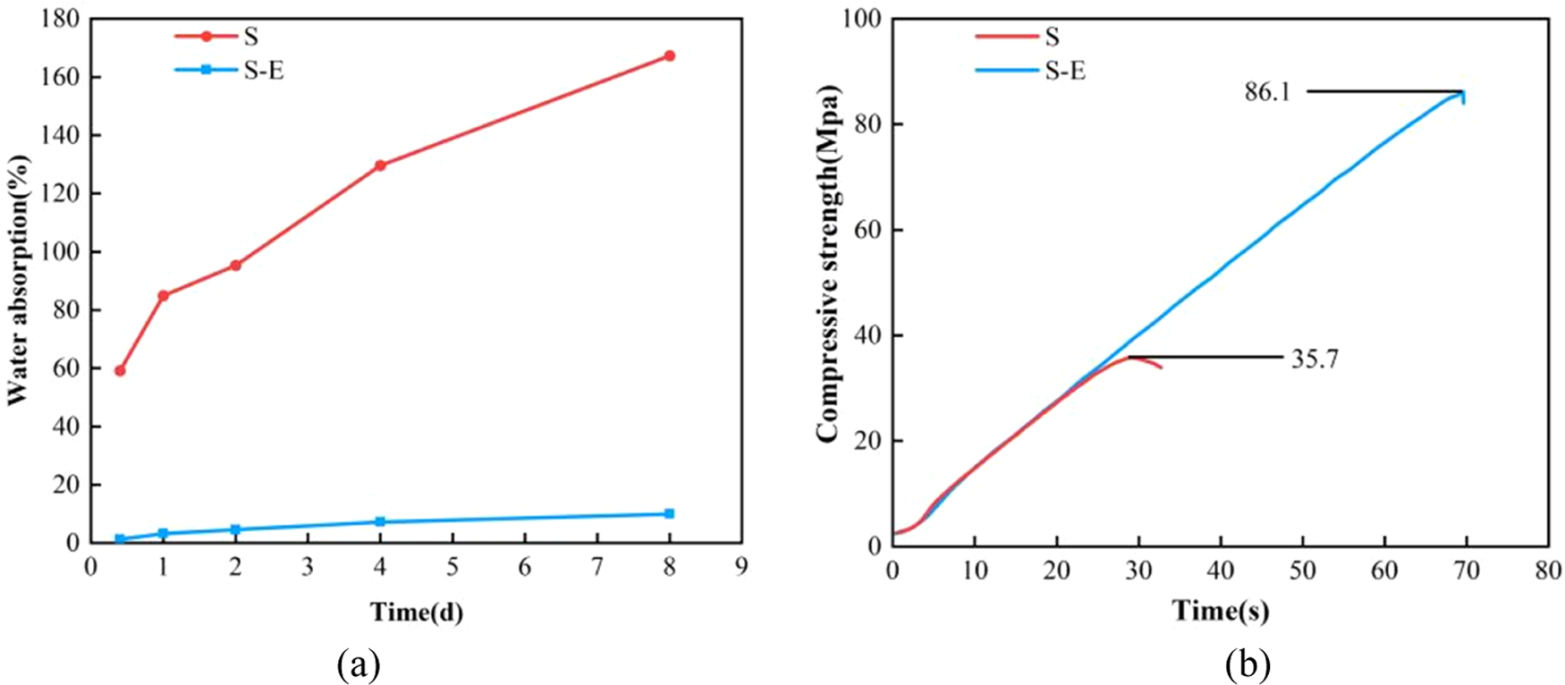
(a) Water absorption rate–time curves of modified woods. (b) Compressive strength–time curves of modified woods.

As the CO-UPR resin is impregnated and cured inside the wood, the water resistance and mechanical properties of the modified wood are also improved. After 8 days of soaking, the water absorption rate of the untreated spruce stabilized at 167.3 ± 1.34%, while the water absorption rate of the modified spruce was 20% below (Figure 6a). The compressive strength of the modified wood also increased from 35.7 ± 0.14 to 86.1 ± 3.25 MPa (Figure 6b).

**Figure 7:**
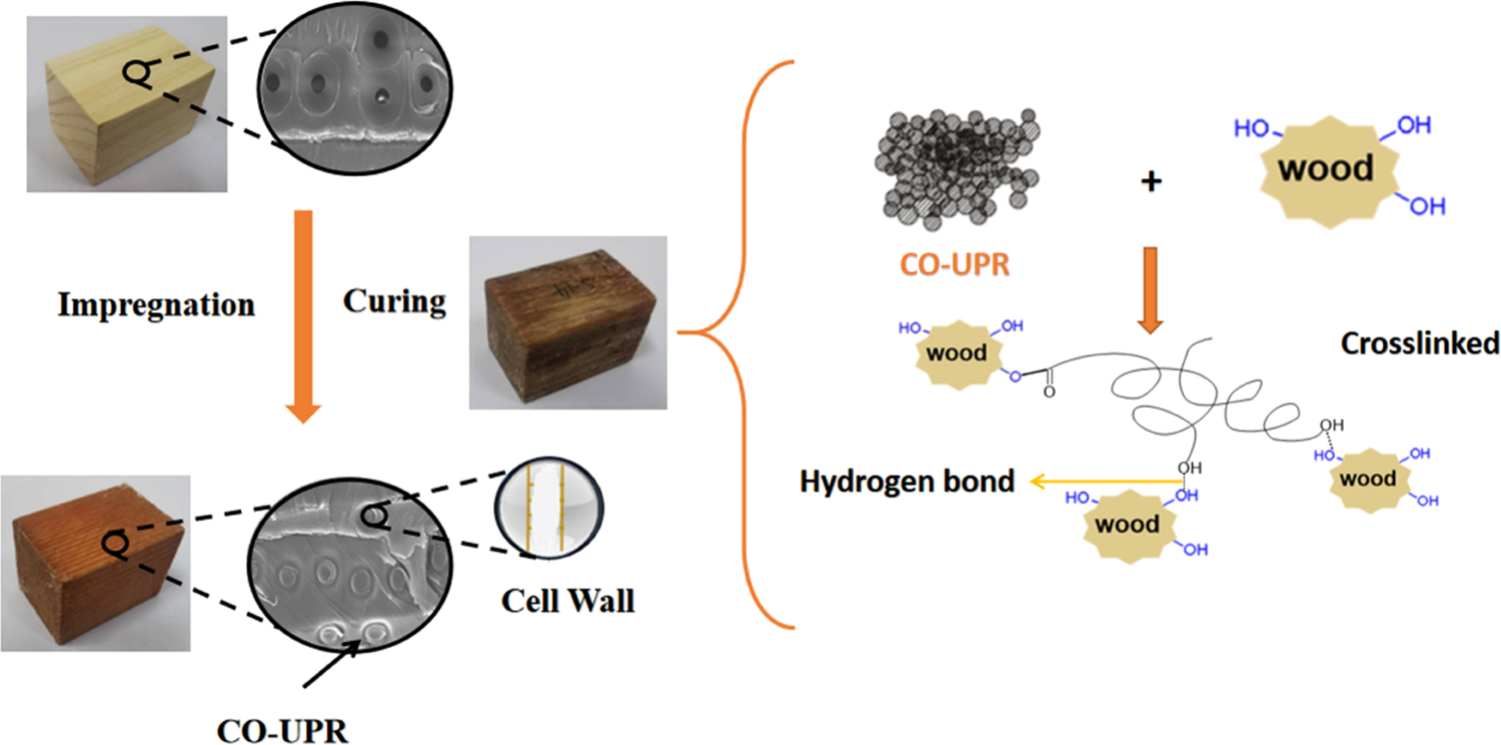
Schematic diagram of the impregnation and curing reaction of the CO-UPR system inside wood.

Figure 7 provides a schematic representation of the impregnation and curing of COEGMA components within the wood structure. The intercellular spaces and lumens of the wood structure are the main pathways for the impregnation and transport of substances into wood cells. For small-sized components, such as short chains or nanoparticles, they can further penetrate the nanoscale pores of the cell walls. With the help of vacuum and pressure treatments, COEGMA containing carboxylic and hydroxyl groups and DVB and PETA containing double bonds can be impregnated into the wood structure. When heated at a given temperature, COEGMA is prone to cross-linking with double bonds, and some carboxylic groups tend to react with the hydroxyl groups in the wood, forming hydrogen bonds with the hydroxyl groups in the wood. This is not only evident in our study but has also been reported by other researchers.^46−49^ The firmly fixed CO-UPR resin network within the wood is expected to have a positive impact on wood modification. Indeed, this is one of the reasons why chemical impregnation was chosen as the method for wood modification in this study.

#### 3.5.2 XPS Analysis of Modified Wood

By detecting the changes in the absorption peak of the C 1s electron sublevel on the surface of modified wood, the surface chemical composition can be analyzed based on the intensity and chemical shift of the C 1s absorption peak.

**Figure 8:**
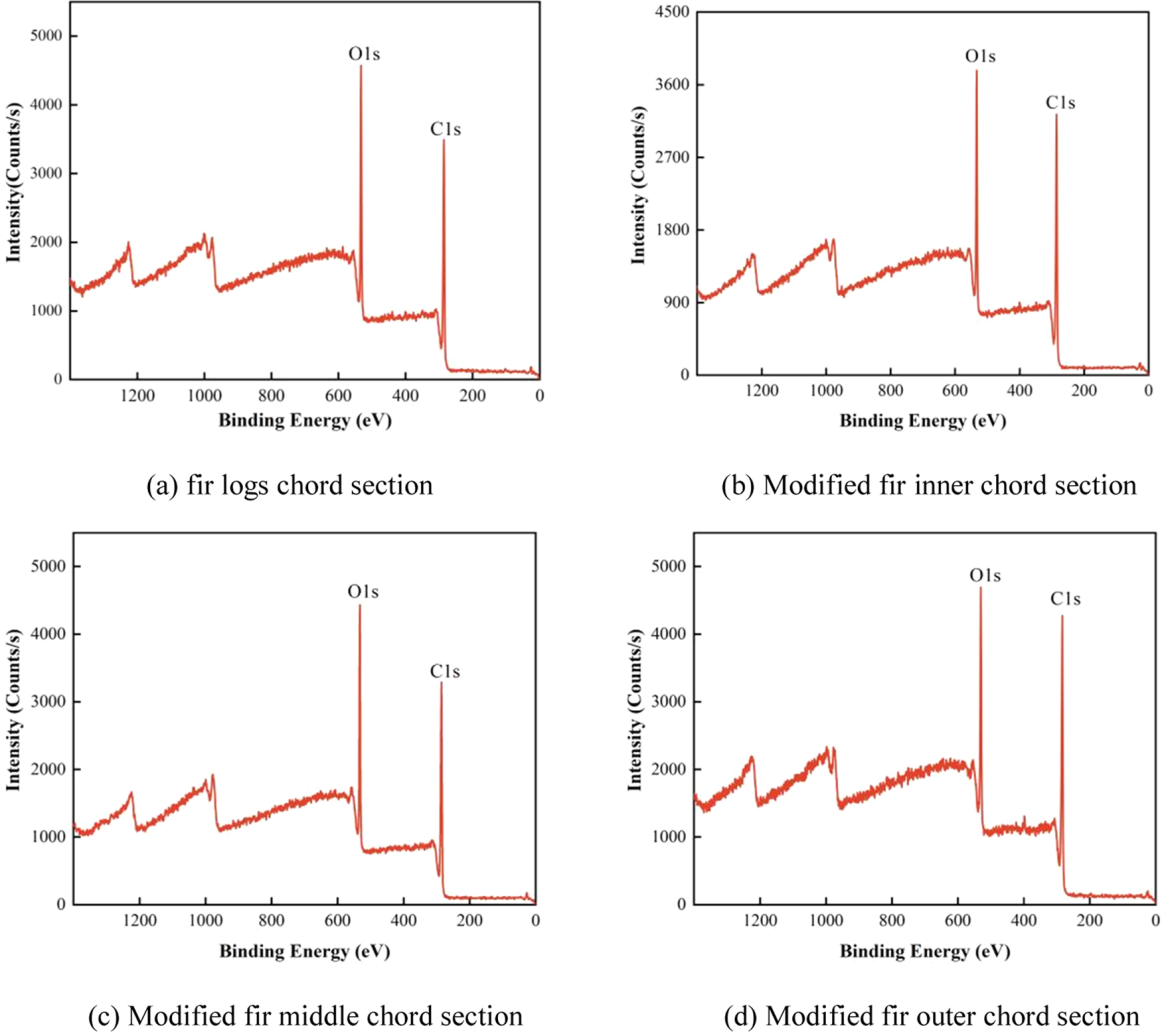
Survey XPS spectra of Chinese fir log and CO-UPR-impregnated modified Chinese fir samples.

**Figure 9:**
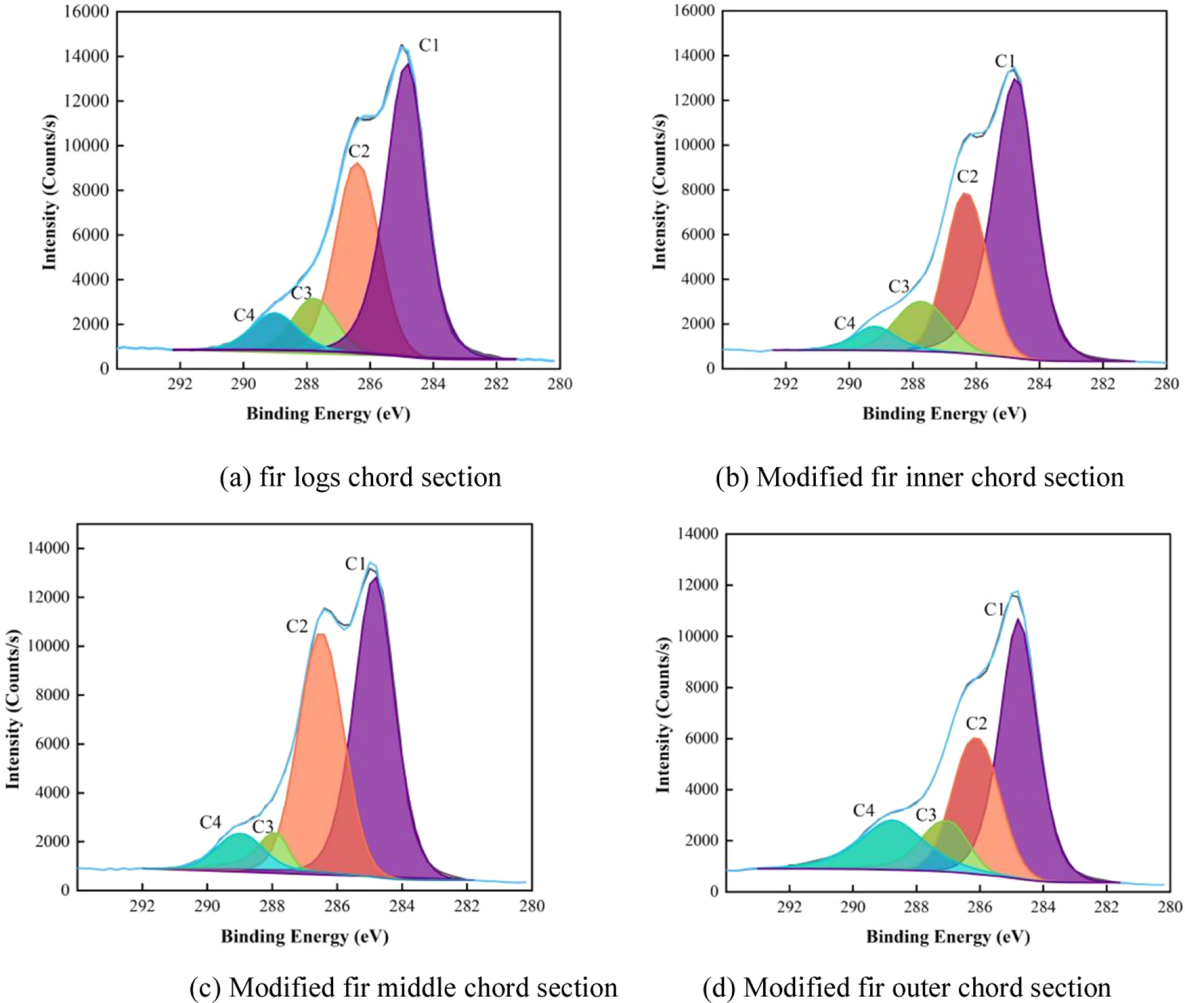
High-resolution C 1s XPS spectrum of Chinese fir log and CO-UPR-impregnated modified Chinese fir samples.

From the XPS full scan spectrum and C 1s high-resolution spectrum (Figure 8), from the observed data, prominent absorption peaks are evident within the binding energy range of 283–290 and at 532 eV. This implies that both the unaltered and the modified Chinese fir surfaces are rich in carbon (C) and oxygen (O) elements. The combination state of C atoms in wood can be divided into four forms.^50,51^ In the C1 component of the spectrum (Figure 9), the C atoms correspond to aliphatic and aromatic carbon chains, only binding to C or H atoms, with an electron binding energy of approximately 284.8 eV. This mainly represents the lignin with the benzyl propane structure in the log and the main chain structure of UPR. The C2 and C3 components correspond to the C–O structure and the binding of C atoms to two noncarbonyl O atoms or one carbonyl O atom, with electron binding energies of approximately 286.4 and 288 eV, respectively. This binding state represents the chemical structure of cellulose and hemicellulose in wood, as both contain a large amount of C atoms linked to hydroxyl groups.^52^ The C4 component corresponds to the binding of C atoms to one noncarbonyl O atom and one carbonyl O atom, with a binding energy of approximately 289 eV. It has a higher oxidation state and produces a larger chemical shift, mainly representing fatty acids, acetic acid, and ester groups in wood extractives and unsaturated polyester resin.

**Figure 10:**
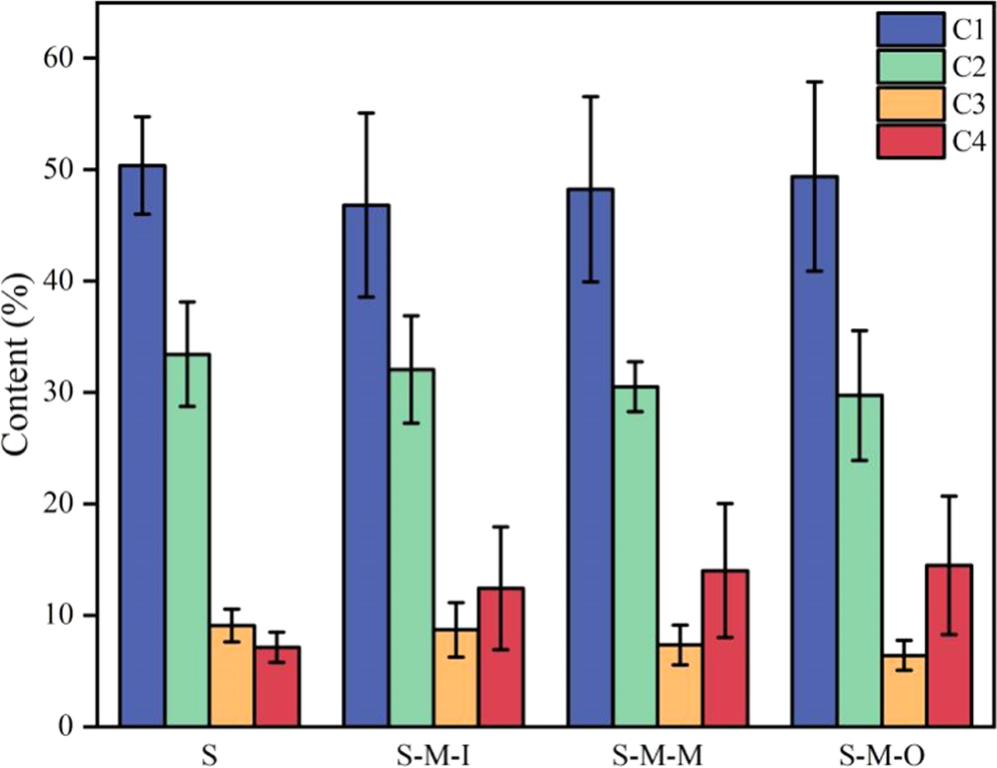
Error analysis chart of original fir wood and fir wood samples modified by the CO-UPR impregnation.

Table 4 shows the relative content changes of different carbon components in the inner, middle, and outer tangential sections of the original and modified wood samples. It can be seen that the relative contents of C1 and C4 components gradually increase, while the relative contents of C2 and C3 components gradually decrease. At the same time, the total oxygen content and the O/C ratio show a downward trend from the inner to outer tangential sections of the original and modified wood samples. This data indicates that compared to the fir logs, the relative content of cellulose and hemicellulose on the surface of modified wood shows a decreasing trend from the inner to outer tangential sections. The reason for this change is that the content of the C1 and C4 components gradually increases in this gradient direction. From Figure 10, it is observed that the error in the various components of the original fir wood is relatively small compared to the modified wood. Due to different degrees of resin impregnation in the modified wood, the errors in the C1 and C4 components are relatively larger compared to those in other components. A comprehensive analysis indicates that the CO-UPR impregnation method mainly relies on lateral penetration, and the degree of impregnation decreases from the outer to inner sections.

**Table 4:** Contents of Chemical Elements on the Surface of Chinese Fir Log and Modified Chinese Fir Samples

sample serial number	C (%)	C1 (%)	C2 (%)	C3 (%)	C4 (%)	O (%)	O/C (%)
fir log chord section (S)	71.99	50.38	33.44	9.06	7.13	28.01	38.90
modified fir inner chord section (S-M-I)	70.97	46.82	32.07	8.69	12.42	29.09	40.98
modified fir middle chord section (S-M-M)	72.38	48.25	30.52	7.33	14.01	27.62	38.15
modified fir outer chord section (S-M-O)	75.32	49.39	29.72	6.40	14.48	24.68	32.76

#### 3.5.3 SEM Analysis of Modified Wood

**Figure 11:**
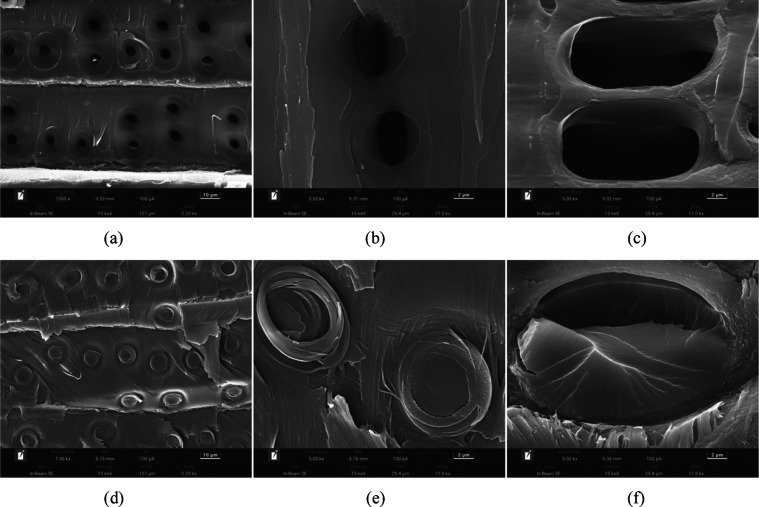
SEM images of Chinese fir logs and samples of Chinese fir impregnated with CO-UPR: panels (a)–(c) in the figure are SEM images of the inner chord section of fir logs, and panels (d)–(f) are SEM images of the modified fir inner chord section. (a), (d) Magnified 1000 times; (b), (c), (e), and (f) magnified 5000 times.

SEM images of the cross sections of the inner tangential surface of the fir logs and modified wood W–S–P are shown in Figure 11. The images of the log cross section reveal that the vessels, wood fibers, and ray cells are free of any obvious attachments and the pit channels are unobstructed. This indicates that CO-UPR can penetrate into the wood interior to accomplish wood modification. In contrast, the modified wood cross section shows that the vessels, ray cells, and wood fibers are filled with and adhered to the resin and the pit channels are completely filled with resin. This indicates that CO-UPR has been successfully impregnated into the wood interior and reacted with small molecules inside the wood without damaging the wood’s microstructure.

Pit channels are pathways for small molecules such as water to enter the wood interior and flow through various microscopic tissue cells. CO-UPR resin fills most of the pit channels inside the wood, blocking water and other small molecules from penetrating, thus greatly improving the water absorption resistance. In addition, the resin that fills the wood fibers and other cells in the wood reacts with and cross-links or forms hydrogen bonds, thereby enhancing the wood’s compressive strength.^25^

#### 3.5.4 Thermogravimetric Analysis of Modified Wood

**Figure 12:**
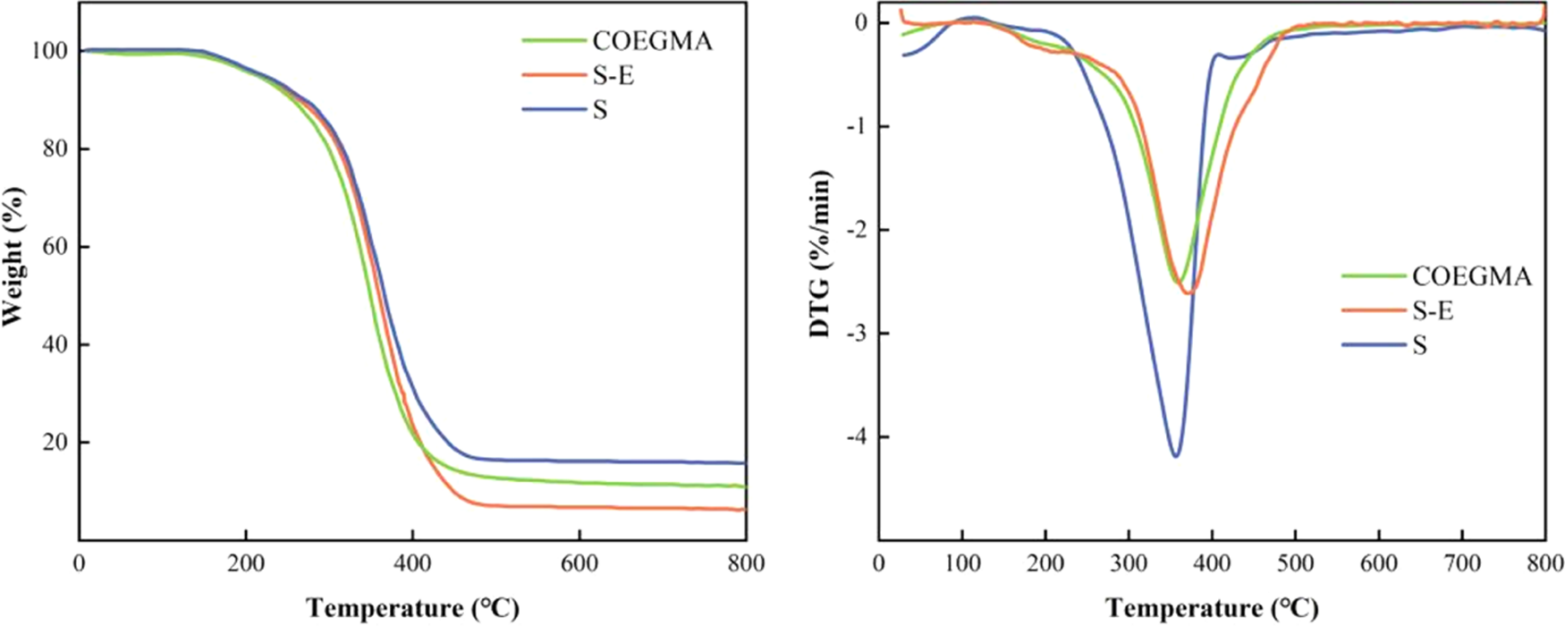
TGA and DTG curves of modified wood, cured COEGMA, and logs.

The TG and DTG curves of Chinese fir logs, modified wood, and cured COEGMA are shown in Figure 12. All three samples exhibit a significant weight loss process. Upon further investigation, it was found that the cured COEGMA shifted toward higher temperatures compared to the untreated wood, while the modified wood shifted toward higher temperatures compared to the cured COEGMA. The thermal analysis results for the untreated and modified wood are listed in Table 5.

**Table 5:** Thermogravimetric Analysis Results of Logs and Modified Woods

	the stage 1		the stage 2		the stage 3		the stage 4	
sample name	temperature range (°C)	weight loss rate	temperature range (°C)	weight loss rate (%)	temperature range (°C)	weight loss rate (%)	temperature range (°C)	weight loss rate (%)
S	30–121	2.02	121–195	1.51	195–396	80.73	396–800	9.97
S-E	30–126	0.12	126–238	5.24	238–468	84.96	468–800	1.53

According to the data in the table, the weight loss process of the fir logs and modified wood can be divided into four stages. The first stage is the dehydration stage, which is mainly caused by the evaporation of residual moisture in the wood cell wall. The modified wood exhibits a notably reduced weight loss rate in comparison with the original log. This suggests that the modified wood absorbs a lesser amount of water, showcasing superior water-resistant properties when subjected to identical treatment procedures and storage conditions. The second stage is characterized by a lower weight loss rate in the fir logs, while the weight loss rate of the modified wood from 126 to 238 °C is 5.24%, which is due to the poor stability of the uncured UPR oligomers in the cured UPR, resulting in weight loss.^33^ The third stage is the thermal decomposition stage of cellulose and hemicellulose in the wood.^53^ The weight loss rate of the log from 195 to 396 °C is 80.73%, while the modified wood involves the decomposition of CO-UPR, with a weight loss rate of 84.96% from 238 to 468 °C. It is worth noting that the modified wood has a higher thermal decomposition starting temperature than the log because the protection of CO-UPR delays the pyrolysis process of the wood components and increases the thermal stability of the wood by about 40 °C.^54^ In the fourth stage, the remaining materials in the original and modified woods continue to decompose until carbonization.

The thermal decomposition of the cured UPR resin can be explained by three main steps. The initial decomposition is observed before 220 °C, where the weight loss is about 6 wt % due to the evaporation of moisture and uncured materials on the surface of the resin.^55,56^ Then, a sharp weight loss is observed between 250 and 490 °C, with a maximum DTG peak at around 360 °C. This is mainly due to the rapid decomposition and volatilization of the cured UPR resin at high temperature until the TGA and DTG curves return to their original baselines. Above 500 °C, a gradual reduction in weight is observed as the heat-stable char produced during the decomposition process slowly oxidizes.

## 4. Conclusions

This study adopts the concept of green chemistry to synthesize a novel castor oil-based unsaturated polyester resin (CO-UPR) and applies it to wood as a carrier to create a green, novel castor oil-based wood–plastic composite material. The main focus is investigating the influence of the CO-UPR resin on wood properties and the performance of the new material in terms of water resistance, dimensional stability, and thermal stability. The results show that CO-UPR resin can not only penetrate into the micro- and nanostructures of wood but also react with the wood, forming a stable cross-linked network inside the wood. Accordingly, the wood’s water resistance, dimensional stability, and thermal performance are significantly improved. Compared with the fir log, the CO-UPR-treated samples exhibited a 147.3% increase in water resistance, a 141.2% improvement in dimensional stability, and a 40 °C increase in thermal stability. This experiment fully demonstrates that the impregnation of the CO-UPR resin into the wood and in situ reaction can obtain wood with high-dimensional stability, water resistance, and good thermal stability. This not only extended the service life of poplar wood but also broadened its range of applications.
